# Age-associated B-cells across the rheumatoid arthritis continuum: from early immunopathogenesis to comorbidities

**DOI:** 10.3389/fragi.2026.1812225

**Published:** 2026-06-25

**Authors:** Daniel Miranda-Prieto, Ana Suárez, Javier Rodríguez-Carrio

**Affiliations:** 1 Department of Functional Biology, Area of Immunology, Faculty of Medicine, University of Oviedo, Oviedo, Spain; 2 Department of Metabolism, Basic and Translational Research on Inflammatory Diseases, Instituto de Investigación Sanitaria del Principado de Asturias (ISPA), Oviedo, Spain

**Keywords:** age-associated B-cells, arthralgia, B-cells, comorbidities, early arthritis

## Abstract

Age-associated B cells (ABCs) are a B-cell subset with distinct transcription and functional properties. Their functions include antibodies secretion, cytokine production, antigen presentation and T-cell stimulation. ABCs have been found expanded in aging, infections and autoimmune diseases such as systemic lupus erythematous, multiple sclerosis and rheumatoid arthritis (RA). ABCs have emerged as plausible mediators of autoimmunity by virtue of their ability to produce autoantibodies and proinflammatory cytokines, as well as trigger T-cell activation, although mechanisms may differ across diseases. Moreover, ABCs may represent a mechanistic link between immunosenescence, premature aging and disease outcomes. Herein, we describe the current knowledge of ABCs in RA with a special focus in the early stages and comorbidity development. Accumulating evidence from preclinical and clinical studies with RA patients have demonstrated disturbances within the ABCs pool. Animal models suggest that ABCs play an important role already in the early phases of the disease and data from RA patients seem to support this hypothesis. Besides, RA risk factors and disease activity may be linked to ABCs expansion, which is associated with high levels of proinflammatory cytokines and correlated with skewed helper T-cell profiles, potentially contributing to inflammaging. Moreover, ABCs may not only be involved in the RA immunopathology, but also in the clinical expression of this disease at synovial and systemic levels. Potential roles of ABCs in cardiovascular, lung, neurological, kidney and metabolic comorbidities are identified, although level of evidence is heterogeneous and limited in some cases. Future perspectives for research, clinical translation and therapeutic implications are discussed.

## Introduction

Age-associated B cells (ABCs) are a rare memory B-cells subpopulation, which can be found in blood and lymphoid organs (Naradikian et al., 2016; Mouat et al., 2022b). They appear in mid-life in healthy individuals and mice, and their splenic and circulating pools tends to increase naturally and steadily with ageing. Furthermore, ABCs were also found to be expanded in other situations such as autoimmunity or intracellular infections (Naradikian et al., 2016; Cancro, 2020; Mouat et al., 2022b). This review aims to provide an integrated overview of ABCs in rheumatoid arthritis (RA), with a specific emphasis on their role across the disease continuum from early disease stages to established synovitis and comorbidity development. We also seek to contextualize ABCs biology within the framework of immunosenescence and inflammaging in RA, and discuss their potential relevance as biomarkers and therapeutic targets, as well as to identify main research gaps in this field.

## Methods

Literature was searched in PubMed between November 2025 and January 2026 using the following keywords: age-associated B-cells, rheumatoid arthritis, inflammatory arthritis, autoimmunity and comorbidities, in different combinations. Only English-language, peer-reviewed articles were included. Additional literature was identified through hand-searching relevant journals and checking reference lists of key review articles (backward citation searching). For the purpose of this review, ABCs were considered as B-cells (CD19^+^) that either express myeloid markers (CD11c) or exhibit T-bet expression, or a combination of thereof, in the presence or absence of CD27, according to the literature (Sanz, 2025). This classification served as an inclusion criterion for literature search and was not meant to be interpreted as a general phenotype definition. For studies referring to alternative phenotypes, their exact definition was explicated in the review and discussed accordingly. Literature outputs were summarized and presented in a narrative format. Figures and tables were generated accordingly, in the light of the review aims, and not intended to be systematic.

## ABCs phenotype, origin and differentiation

**FIGURE 1:**
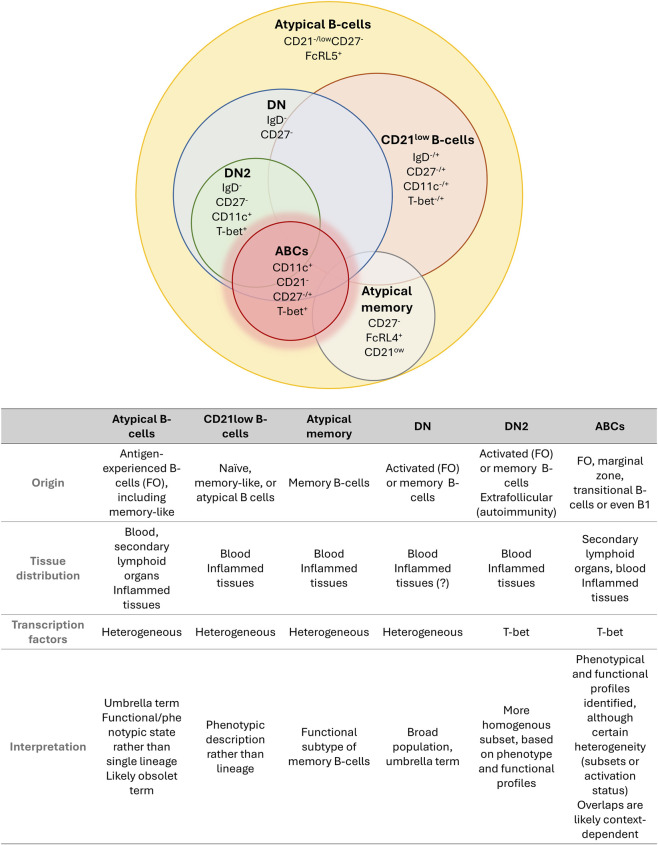
ABCs and related B-cell subsets. Schematic representation depicting overlaps among ABCs and related B-cell subsets (atypical B-cells, atypical memory, DN, DN2 and CD21low B-cells) (top) and similarities/differences in origin, main roles, and subset interpretation (bottom). The borders of the ABCs population are indicated as blurry as the extent of phenotype overlap may be context-dependent. Venn diagram and table are not derived from a systematic analysis and should be considered as general evidence for the purpose of this review.

ABCs were initially identified by the expression of T-bet^+^, CD21^low/-^, CD11c^+^, CD11b^+^ within B-cells (Mouat et al., 2022b), mostly lacking CD27 and IgD expression (Sanz, 2025), although there is a large heterogeneity in the markers that define ABCs in the literature. Those termed atypical, double negative 2 (DN2), or CD21^low^ B-cells have also been described as similar populations to ABCs, although current evidence suggest that ABCs are likely an independent subset within DN2 (Figure 1). Phenotypical, functional, metabolic, and anatomical studies have strengthened this observation (Figure 1). In this regard, atypical B-cells were initially identified in pathological contexts as B-cells lacking CD27 and/or CD11c expression, although current evidence suggest that this term is obsolete (Sanz, 2025). CD21low B-cells represents a broader phenotypic category that can include several activated or tissue-like memory subsets (Keller and Warnatz, 2023). DN are a heterogeneous population, defined by the absence of IgD and CD27 expression, where the DN2 subtype is hallmarked by T-bet expression and exhibits a phenotypical, functional and pathological overlap with ABCs in humans (Lamprinou et al., 2026). However, differences were noted especially regarding CD27 and class-switched Ig isotypes expression by ABCs, Moreover, tissue distribution and potential precursors largely differ (Figure 1). Overall, some of these populations may be considered as phenotypical or functional definitions (Figure 1), rather than representing single lineages. Importantly, the role of (disease) context seems to be critical to understand for the accurate delineation of these cellular subpopulations (Lamprinou et al., 2026), which challenges the establishment of firm boundaries around the ABCs population.

ABCs display a distinct transcriptional signature from other B-cell subsets, such as naïve or memory B-cells (Holla et al., 2021; Winslow and Levack, 2025). Regarding cell surface markers, these cells were characterized by mature B-cell markers (CD19, CD20, CD21), and upregulation of costimulatory molecules (CD80, CD86) and inhibitory receptors (FCGR2B, LILRB2). Regarding transcription factors, ABCs show higher expression of T-bet*,* the master regulator of ABCs, and AICDA, a gene involved in somatic hypermutation and class-switching. ABCs are also hallmarked by a low CXCR5 expression (Holla et al., 2021; Vidal-Pedrola et al., 2023). Also, plasma cell differentiation genes (PRDM1, BLIMP1 and XBP1) were highly expressed in ABCs compared to naïve B-cells, but to a lower level compared to their memory counterparts (Vidal-Pedrola et al., 2023). Equivalent differences were noted for cytokines and proteins involved in motility and migration (Holla et al., 2021). As antigen-experienced B-cells, ABCs show lower proportions of cells expressing IgM and IgD than naïve B-cells. Similarly, the mutation of BCRs in ABCs appear to be in the mid-range between naïve and germinal center B-cells (Ma et al., 2019), but demonstrating significant somatic hypermutation derived from antigen stimulation overtime (Russell Knode et al., 2017). Despite these common features, it seems that different ABCs markers and transcriptional profiles might vary depending on differentiation stages, subset plasticity or disease background (discussed later). For instance, ABCs in malaria showed higher NR4A1 and lower BATF expression, whereas ABCs from HIV patients displayed an opposite tendency (Holla et al., 2021).

ABCs may derive from several potential peripheral progenitors such as follicular (FO) and potentially also from marginal zone (MZ) preimmune B-cells, transitional or B1 B-cells (Cancro, 2020) rather than deriving from bone marrow precursors. The differentiation of these B-cells into ABCs needs the engagement of Toll-like receptors 7 or 9 (TLR7/9) with internalized antigens from intracellular pathogens or endogenous antigens (chromatin or dead cell fragments) by B-cell receptor (BCR) (Cancro, 2020; Mouat et al., 2022b). Despite this step being crucial, subsequent cytokine stimulation is necessary to induce naïve B-cells into ABCs fate. IFN-γ or IL-21 are the main cytokines that participate in ABCs differentiation by promoting T-bet expression, but IL-12 and IL-18 can be also involved (Cancro, 2020; Mouat et al., 2022b). The ABCs differentiation may be CD40-dependent, through the interaction with helper T-cells, especially follicular (Tfh) and peripheral (Tph) helper T-cells (Cancro, 2020; Mouat et al., 2022b; Li et al., 2023). Cognate T-cell help is required for ABCs generation, as MHC-II or CD40-deficient FO B-cells did not result in ABCs generation (Russell Knode et al., 2017). Importantly, T-cells are also critical likely due to their provision of cytokines, including IFN-γ (Levack et al., 2020). However, exposure to IFN-γ from bystander cells may also promote the ABCs phenotype (Cancro, 2020). Interestingly, ABCs can arise intra- or extra-follicularly (Viant et al., 2021), and recent evidence have reported ABCs formation without germinal center entry (reviewed in (Mouat et al., 2022b). In fact, T-cell/B-cell interaction is especially relevant in chronic inflammatory settings, where repeated antigenic stimulation and sustained T-cell collaboration may promote extrafollicular B-cell responses.

Collectively, a unique signalling triad encompassing BCR signalling, endosomal nucleic acid sensor ligation and type-I inflammatory cytokine milieu underlie ABCs generation and may be the missing link connecting ABCs expansion with aging, infection and autoimmunity. Importantly, ABCs fail to exhibit proliferation and activation by BCR cross-linking alone, whereas TLR-7/9 exhibited robust proliferative responses. In contrast to other B-cell pools, ABCs show both BlyS/BAFF receptors, BR3 and TACI. Although ABCs survival is not dependent on BLyS/BAFF, they can compete with other B-cell subsets, such as follicular (FO) and marginal zone (MZ) B-cells, and potentially outcompete them within shared niches (Cancro, 2020; Mouat et al., 2022b). This ‘scavenging’ effect leads to an accumulation of antigen-experienced cells, likely chronically activated, at the expense of the naïve counterparts, hence leading to a skewed B-cell pool, which may account for their effect on immunosenescence impairments during aging.

At the metabolic level, ABCs exhibit higher oxygen consumption and extracellular acidification rates, thus suggesting enhanced oxidative phosphorylation and anaerobic glycolysis usage than FO B-cells (Frasca et al., 2021a). These differences were larger in aged mice, also pointing to age-driven changes in function in addition to levels in ABCs pools. These findings confirm a hypermetabolic state in ABCs, which is in line with their higher expression of the Glut1 transporter (Frasca et al., 2021a). Further analysis from public databases confirmed these findings in human ABCs (Han et al., 2023). This hypermetabolic status may be related to their sustained, enhanced proinflammatory status, autoantibody production and secretion of cytokines (Frasca et al., 2021a), and parallel immunosenescence markers. IFN-γ played a pivotal role in metabolic rewiring during ABCs differentiation by upregulating the expression of genes involved in glycolysis (Han et al., 2023). Interestingly, T-bet, a downstream transcription factor of IFN-γ and the master transcription factor for ABCs, can impair Bcl-6-dependent repression of the glycolysis pathway gene programme. In fact, glycolysis restriction could impair ABCs formation, and T-bet deficiency in B-cells demonstrated a profoundly amelioration of glycolysis (Han et al., 2023). Taken together, these findings demonstrate that T-bet can be considered a core regulator of ABCs, coupling immune signals and metabolic programming in driving pathogenic ABCs formation.

Anatomically, ABCs are found in blood, spleen and bone marrow, and less frequently in lymph nodes, at least in healthy individuals (reviewed in (Naradikian et al., 2016)). Recent studies have revealed that ABCs are enriched at the T:B border, probably due to altered CCR7 expression (Benson et al., 2008). This location may be central to understand the unique functional profile of ABCs. However, during ongoing inflammation, ABCs can exit lymphoid organs and circulate to inflamed tissues, as observed during infection or autoimmunity (Mouat et al., 2022b). Trafficking, homing patterns, and chemokine receptor expression differed from FO and other recirculating subsets.

### Main functions

TLR7 and TLR9 ligation, either by viral nucleic acids or nucleic acid-containing self-antigens, trigger ABCs activation (Rubtsova et al., 2015; Naradikian et al., 2016; Mouat et al., 2022b) and production of antibodies, involved in viral clearance or autoimmune responses, respectively (Naradikian et al., 2016; Mouat et al., 2022b). It has been demonstrated that ABCs can undergo differentiation into plasma cells and produce antibodies, namely, IgG1 in humans and IgG2a/c in mice (Naradikian et al., 2016; Mouat et al., 2022b). Antibody production and secretion by ABCs have been also confirmed in different mice models (Mouat et al., 2022b). In lupus-prone mice model SLE-NZB/WF1, ABCs were able to produce anti-chromatin IgGs (Rubtsov et al., 2011). Besides, in SLE MER^−/−^ model, knocking out TLR7 caused a reduction in anti-chromatin IgG autoantibodies (Rubtsov et al., 2012). T-bet deletion in B-cells shifted the isotype distribution towards lower IgG2a and higher IgG2b and IgG1 levels compared to control mice (Rubtsova et al., 2017). Importantly, ABCs may also play a role in the germinal center response, due to their T-bet expression (Rubtsova et al., 2017), which is critical for the production of autoreactive B-cell clones, and can in turn exacerbate autoimmune disease (Mouat et al., 2022b). Furthermore, ABCs might be a specialized memory B-cell subset in the elderly, acting against chronic infections or endogenous pathogens or cellular debris (Rubtsova et al., 2015).

Additionally, ABCs can serve as effective antigen presenting cells (APCs), by virtue of their high levels of MHC-II, CD80 and CD86, together with higher expression of genes involved in vesicular transport, cytoskeletal rearrangements and higher levels of CD11c (reviewed in (Ma et al., 2019)). ABCs can thus participate in CD4^+^ T-cell differentiation, especially to Th1 and Th17 (Ma et al., 2019). They can also secrete cytokines (IFN-γ, IL-4, IL-6 and IL-10) upon TLR-7/9 activation, which may be relevant in shaping T-cell responses.

ABCs secrete a diversity of cytokines, including IL-4, IL-17, IL-10, IFN-γ and TNF (Naradikian et al., 2016; Mouat et al., 2022b). Divergent cytokine signatures, involving both pathogenic and protective profiles, have been described, and cytokine profiles largely differ in autoimmune diseases (Naradikian et al., 2016; Mouat et al., 2022b). Cytokine production by ABCs likely accounts for skewed responses by T-cells and other cellular components (reviewed in (Mouat et al., 2022b), including sustained B-cell maturation and thwarted B-cell responses, thus leading to broad effects within the immune system architecture.

ABCs can also interact with and present antigens to T-cells, which could also play a key role in autoimmune diseases (Mouat et al., 2022b). ABCs usually locate near T-cell zones and B:T cell border areas in the spleen. Besides, ABCs show an increased expression of CCR7, which facilitates migration to T-cell areas in response to chemokines CCL19 and CCL21. Once in the T-cell areas, ABCs can form stable interactions with T-cells, even and more effectively than follicular B-cells due to their high expression of costimulatory molecules (Ma et al., 2019; Mouat et al., 2022b). In fact, *in vitro* and *in vivo* studies have demonstrated a more robust activation of T-cells in the presence of ABCs (Liu et al., 2015; Wang et al., 2016; Rubtsova et al., 2017), especially in autoimmune patients (reviewed in (Li et al., 2023)).

Altogether, these bidirectional interactions suggest that ABCs are not passive recipients of T-cell help for their differentiation, but active partners in a (pathogenic) feedback loop. By presenting antigen and delivering costimulatory signals, ABCs may reinforce T-cell polarization toward inflammatory phenotypes, thereby amplifying cytokine production which in turns further sustains ABCs differentiation and activation. In this sense, T-cell/B-cell crosstalk may help maintain an extrafollicular inflammatory circuit that may be particularly relevant in autoimmunity, where persistent immune activation can occur both in secondary lymphoid tissues and at the target tissue level.

### ABCs and immunosenescence and inflammaging

Immunosenescence refers to the age-related remodelling of the immune system, characterized by a decline in adaptive immune competence, shifts in lymphocyte composition pools, reduced antigenic responsiveness, and the accumulation of functionally altered, chronically-activated immune cells (Caruso et al., 2022). In parallel, inflammaging describes the persistent, low-grade inflammatory milieu that develops with aging and shapes both innate and adaptive immune responses, thereby contributing to tissue dysfunction and increased disease susceptibility (Santoro et al., 2021). These intertwined processes are increasingly recognized as hallmarks of RA, where they may favour loss of immune tolerance, sustained cytokine production, and the transition from localized synovitis to systemic inflammation (Chalan et al., 2015; Weyand and Goronzy, 2025). Notably, features consistent with immune aging have been reported in RA unrelated to calendar age, suggesting that accelerated immunological aging may be part of the disease process (Raza et al., 2025; Weyand and Goronzy, 2025). In fact, immunosenescence traits have been reported already in early stages, including the arthralgia setting (Raza et al., 2025), hence pointing to a role in disease initiation and progression, rather than a consequence or epiphenomena.

Although initially linked to aging by their age-dependent accumulation, ABCs have been revealed as active drivers of immunosenescence as well. ABCs may impair B-cell homeostasis by promoting TNF-dependent pre-B-cell apoptosis in the bone marrow, and displacing follicular B-cells in secondary lymphoid organs through preferential BAFF usage. Consequently, ABCs expansion can result in skewed B-cell pools and ultimately impair the ability of naïve B-cells to mount responses against new antigens (Cancro, 2020; Santoro et al., 2021). In parallel, recent studies have uncovered novel mechanisms by which ABCs may contribute to T-cell aging, thereby pointing to their role as critical mediators driving age-associated adaptive immune dysfunction also within the T-cell compartment (Khan et al., 2026). These effects broaden the relevance of the B-cell/T-cell bidirectional interaction in shaping ABCs pathogenicity and role in disease mechanisms. This process may be particularly relevant in RA, where senescent CD4^+^ T-cells have been involved in disease pathogenesis (Rodríguez-Carrio et al., 2015; Gao et al., 2022). Within this framework, ABCs emerge as a plausible link to understand the connections between immunosenescence, inflammaging, and RA from a functional perspective at the whole immune system level.

## ABCs in RA

There has been an exponential grow in ABCs research in recent decades, reaching up to 221 publications in the last 5 years (as of January 2026), out of a total of 431 results from 1976 to 2026. Globally, these mostly belong to thematic areas such as viral infections, aging, cancer, and autoimmunity. Despite the growing evidence supporting a role for ABCs in autoimmunity, preclinical and general studies overall predominated. The current, fragmented literature landscape may jeopardize the understanding of disease-specific mechanisms, prevent clinical translation, and may obscure the identification of therapeutic avenues. In the next sections, we will summarize what is known pertaining the participation of ABCs in RA, and we will discuss how these may hallmark the different clinical stages of RA, from disease triggering to clinical manifestations.

Literature search refinement to the to the terms “age-associated B cells” and “rheumatoid arthritis”/“arthritis” revealed a significant number of publications addressing the analysis of ABCs subset in RA, from animal and experimental studies to clinical cohorts (Table 1).

**TABLE 1:** Summary of studies on ABCs in RA populations**.**

Author, year	N	Platform	ABCs phenotype	Source	Disease stage	Serostatus	Treatment status	Main results
Human studies								
Bao et al. (2022)	75 RA 27 HC	Flow cytometry	CD19^+^CD11c^+^T-bet^+^	Blood (PBMCs)	Established	76% anti-CCP 76% RF	Unspecified	• Increased in blood in RA • Associated with DAS28 • Associated with proinflammatory cytokines (IFN-γ, IL-21 and IL-10) • Lower ABCs levels associated with good response to treatment (unspecified DMARDs) • Differential expression of miR-142 and miR-146, favoured ABCs differentiation (combined with IL-21 and IFN-γ)
Dai et al. (2024)	6 RA upon treatment (tofacitinib)	Flow cytometry and single-cell RNAseq	CD27^−^IgD^-^CD11c^+^CD21^−^T-bet^+^	Blood (PBMCs)	Established	Unspecified	100% TOFA	• Tofacitinib (4 weeks) reduced circulating ABCs levels • Tofacitinib inhibited human ABCs differentiation *in vitro*
Rubtsov et al. (2011)	26 RA 14 SSc 13 SLE 36 HC	Flow cytometry	CD19^+^CD21^−^CD11c^+^	Blood (PBMCs)	Established	Unspecified	Unspecified	• Increased in blood in RA • Higher increase in female compared to male patients
Qin et al. (2022)	67 RA 23 SpA 25 OA 29 HC	Flow cytometry and RNAseq	CD19^+^CD27^−^IgD^-^CD21^−^CD11c^+^	Blood (PBMCs)	Established	79% anti-CCP+ 61% RF+	32% NSAIDs 57% GC 64% MTX 43% LEF 24% HCQ 3% SSZ 28% bDMARDs + tsDMARDs	• Increased in blood in established RA • Increased in SF compared to blood • Associated with tender joint count, swollen joint count, DAS28 and anti-CCP • Increased production of IL-21, IL-17A, TNF and IL-4 • Upregulated expression of genes related to neutrophil activation, leukocyte migration and adhesion • ABCs functionally activate FLS via TNF
Vidal-Pedrola et al. (2023)	50 early RA 12 established RA 17 PsA 16 HC	Flow cytometry	CD19^+^CD11c^+^CD21^−^	Blood (PBMCs) and synovial (SFMCs)	Early and established	Early 68% anti-CCP + or RF+ Established 92% anti-CCP + or RF+	Early (n = 50): Untreated Established (n = 12 100% MTX 20% HCQ	• Increased in synovium in early RA • Differential gene expression profiles in ABCs (interleukin receptors, adhesion molecules, and chemokine receptors) • Different phenotype between ABCs from blood and synovium (FcRL and chemokine receptors)
Kim et al. (2025)	RNAseq 12 RA 4 HC Flow cytometry 50 RA 11 HC	RNAseq and flow cytometry	CD19^+^CD11c^+^ T-bet^+^	Blood (PBMCs) and synovial (SFMCs)	Early	Untreated 91% anti-CCP+ 83% RF+ Treated: 93% anti-CCP 84% RF+	Untreated (n = 36) Treated (n = 44) 90% GC 81% MTX 18% LEF 18% SSZ 56% HCQ 22% IS	• Increased in early RA in blood compared to HC and treated RA group • Associated with ESR, CPR, swollen joint counts, and DAS28 • Potential biomarker of disease activity • DMARDs usage for 3–6 months reduced the proportion of ABCs in paired samples • Association with TNF and IL-10 levels • Differential expression of pathways related to phagocytosis and antigen processing and presentation
Miranda-Prieto et al. (2025)	58 RA 11 CSA 33 HC	Flow cytometry	CD19^+^CD21^−^CD11c^+^	Blood (PBMCs)	Early and CSA	CSA 45% ACPA+ 54% RF+ RA 65% ACPA 68% RF+	Untreated	• Increased in blood in early RA, subtle increase in CSA • Associated with proinflammatory mediators (IFN-γ, TNF, IL-6 and IL-21) • Higher ABCs levels were associated with poor therapeutic outcomes upon csDMARD (GC + MTX) at 6 and 12 months • Associated independently with atherosclerosis presence and plaque number • ABCs were connected to proteomic signatures involved in B-cell activation, T-cell responses and cellular pathways related to atherosclerosis development
Zhang et al. (2019)	36 RA 15 OA	RNAseq, mass cytometry and IF	Mass cytometry: IgM^−^IgD^−^HLA-DR^++^ CD20^+^CD11c^+^ IF: CD20^+^ T-bet^+^ CD11c^+^	Synovial tissue	Established	66% anti-CCP+ 66% RF+	38% GC 27% MTX 16% TNFi 2% RTX 5% ABA 8% TOFA	• Increased in synovium in established RA • Three putative subsets identified • Increased expression of interferon stimulated genes (GBP1 and ISG15) in ABCs
Dunlap et al. (2024)	12 RA	Single-cell RNA seq and flow cytometry	CD19^+^CD11c^+^FCRL5^+^ T-bet^+^ZEB2^+^	Synovial tissue	Established	75% anti-CCP + or RF+	25% MTX 25% TNFi 25% LEF 8% SSZ 16% uspecified	• ABCs are enriched in synovium in RA, although more abundant in blood compared to synovium • Increased expression of interferon-stimulated genes (IFI30) • ABCs exhibited higher rates of somatic hypermutation and class-switch recombination
Animal studies								
Qin et al. (2022)	CIA (C57B6/L)	Flow cytometry and RNAseq	B220^+^CD11c^+^Tbet^+^	Spleen, hind paws and blood	​	​	​	• Increased in CIA mice in spleen, blood and joints (not significantly) • ABCs differentiation was promoted by IL-21 and TLR7 in CIA
Mouat et al. (2021)	CIA (C57B6/L)	Flow cytometry	CD19^+^CD11c^+^Tbet^+^	Spleen, synovial fluid cells (knee and ankle joints) and blood	​	​	​	• Increased in the spleen after CIA induction, larger increase in gammaherpesvirus-infected mice • Latent viral infection worsened clinical score

Studies analyzing ABCs, in RA patients (human studies) or mouse models (animal studies) are summarized. Studies are listed by disease stage and tissue sources. Sample sizes, biological sources, phenotype, disease stages, serostatus, treatment usage and main results are indicated. References are indicated in the first column. ABA, abatacept; DMARDs, disease-modifying anti-rheumatic drugs; GC, glucocorticoids; HCQ, hydroxychloroquine; IF, immunofluorescence; IS, immunosuppressive agents; LEF, leflunomide; MTX, methotrexate; RTX, rituximab; SSZ, sulfasalazine; TOFA, tofacitinib.

Overall, there is consistent evidence indicating that the ABCs subset is expanded in RA, both in animal studies as well as in human patients (Table 1). Moreover, certain studies also revealed changes within the pool of ABCs beyond levels, thereby suggesting potential disease-specific mechanisms.

In the setting of RA, ABCs exhibited an activated (CD69) and proliferative (Ki67) profile, also characterized by high levels of costimulatory molecules, and MHC class II (Vidal-Pedrola et al., 2023). Furthermore, ABCs showed higher frequencies of IgG and IgA, as class-switched B-cells. Additional differential traits such as high expression of some inhibitory receptor genes (FCGR2A/C and LILRB1/3) and upregulation of CD97, an adhesion G protein-coupled receptor, were also noted in RA (Vidal-Pedrola et al., 2023). Moreover, Wang and coworkers found comparable transcriptional profiles on CD11c^hi^ B-cells between SLE and RA patients, hence suggesting shared pathogenic mechanisms across conditions, and allowing certain translation from SLE studies (Wang et al., 2018). However, RA studies have also pointed to unique profiles of ABCs in RA. Of note, ABCs exhibited a characteristic profile expression of FcRL members (FcRL2, FcRL3 and FcRL5), compared to naïve and memory B-cells (Vidal-Pedrola et al., 2023). Cytokine secretion patterns were also found to be distinct, with a higher expression of TNF and IL12A and low expression of IL23A, IL6 and IL6ST being retrieved in RA (Holla et al., 2021). RA was also associated with a differential chemokine receptor profile in ABCs. CXCR3 and CXCR3R1 were highly expressed while CXCR4 and CXCR5 were diminished in RA, probably suggesting recruitment and enhanced migration to inflamed synovium, as CXCR4A and CXCR5 are involved in recruitment to lymph nodes (6). Importantly, ABCs in RA also showed heterogeneity based on their location. ABCs from synovial tissue exhibited a more proliferative and secreting profile, with higher frequencies of ABCs expressing FcLR4, IgG and IgA, whereas FcLR3 and FcRL5 were less expressed in comparison with ABCs from peripheral blood (Vidal-Pedrola et al., 2023). These findings demonstrate that ABCs can display unique localization and trafficking patterns, which may be relevant to understand the natural history of RA.

### ABCs as drivers of disease triggering

There is increasing evidence that ABCs may be involved in RA pathogenesis, although their actual contribution is yet to be elucidated, and causal relationships should be considered with caution in the light of the existing evidence (dicussed later). ABCs are known to effectively exert a number of mechanisms which may underlie arthritis development and perpetuation (reviewed in (Ma et al., 2019)), namely, (i) antigen presentation, which may activate autoreactive T-cell clones and shape immune responses, (ii) cytokine production, which may shape immune cell responses and sustain autoreactive clone activation, and (iii) autoantibody production, which may trigger tissue damage and thus, disease manifestations. Of note, the T-cell compartment may determine whether these functions translate into pathogenicity. Tfh and Tph cells are plausible upstream drivers of ABC expansion in early RA, as they provide the cytokines and costimulatory signals needed for ABC differentiation and maintenance. In addition, skewing toward Th1-like responses and accumulation of senescent or chronically stimulated T-cell subsets may strengthen this proinflammatory environment, in turn facilitating ABCs activation, class switching, and antibody production. Moreover, chronic antigen exposure favour dysfunctional, senescent-like T-cell subsets in RA, which may sustain persistent cytokine output and costimulatory interactions that help maintain ABCs expansion in this setting.

**FIGURE 2:**
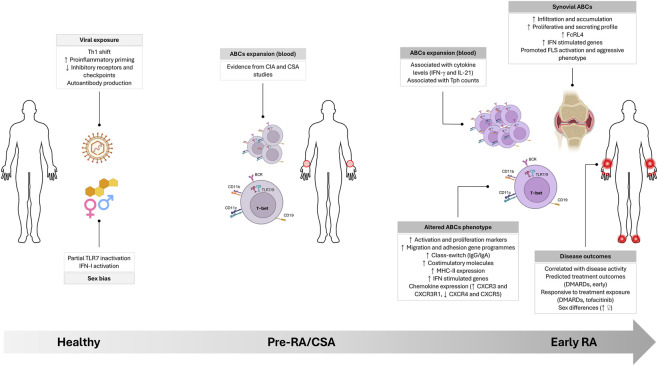
Potential roles of ABCs across the RA continuum, with emphasis on early stages. In healthy individuals, viral or innate immune stimulation (such as, Toll-like receptor-7 activation), or sex-specific stimuli may promote transient ABCs differentiation. During the pre-clinical phase (pre-RA/clinically suspect arthralgia, CSA), ABCs have been found to be increased, supporting their early involvement before arthritis onset. In early RA, ABCs are further increased in blood and associate with inflammatory cytokines and T-cell collaboration. Concomitantly, ABCs accumulate in synovial tissue, where they display an activated, antigen-experienced phenotype and contribute to local inflammation. Phenotypic alterations in early RA ABCs include increased activation/proliferation markers, enhanced migration/adhesion molecules and chemokine receptors (such as, CXCR3, CCR6, CCR7), transcriptional programs (T-bet–related), and pro-inflammatory cytokine production. Clinically, ABC expansion and activation correlate with disease activity, structural damage (synovitis), and therapeutic responses. Overall, evidence supports a progressive dysregulation of ABCs from systemic priming to joint-localized pathogenic functions along the earliest RA stages.

Animal studies have been instrumental in deciphering the disturbances within ABCs pools in RA, especially in the earliest stages, as well as in shaping the understanding between risk factors and RA development. First, ABCs may contribute to sex bias in autoimmunity. ABCs are known to be expanded to a higher degree in females compared with males not only in aging but also during infections or autoimmunity (Rubtsov et al., 2011; Ricker et al., 2021). Mounting evidence has demonstrated a role for TLR7, which may escape from total X-chromosome inactivation (Figure 2). In animal models of lupus, TLR7 was responsible for ABCs accumulation, oligoclonality and differentiation into pathogenic and pro-inflammatory subsets. Importantly, RNA-seq analyses demonstrated that type I interferon (IFN) pathway was enriched in ABCs in females. TLR7 duplication in males recapitulate these observations, and override pathogenic features of ABCs, leading to enhanced haematological and immunological alterations, and resulting in severe pulmonary inflammation and early mortality (Ricker et al., 2021). This reinforced previous evidence on the TLR7 dependency for ABCs accumulation and/or differentiation (Rubtsova et al., 2012), which has been also observed in arthritis models (Qin et al., 2022). Importantly, although sex hormones are known to contribute to sex dimorphism in immune responses, especially for Th skewing profiles and immunoglobulin production, there is no evidence in the literature on their potential effects on ABCs.

Furthermore, ABCs have been reported to expand during viral infections (Figure 2), including Epstein-Barr, cytomegalovirus, hepatitis virus, human immunodeficiency virus, influenza, or SARS-CoV2, among others. Importantly, viral infections are well-established risk factors for RA development (Kudaeva et al., 2019), as well as for other autoimmune conditions such as SLE, MS or SjD. Early viral infections have been demonstrated to correlate with more severe clinical outcomes upon disease onset in animal models of MS or RA (Mouat et al., 2021; 2022c). This effect overlapped with a Th1-shifted profile at the synovial level and a skewed T-cell profile at secondary lymphoid organs, although divergent patterns were noted between spleen and lymph nodes. Knock-out models demonstrated that ABCs ablation abrogated clinical exacerbation of the arthritis model (Mouat et al., 2021), thereby demonstrating the central role of ABCs in this phenomenon. Interestingly, functional studies have revealed that latent viral infections led to a consistent ABCs accumulation, especially in secondary lymphoid organs, and primed TNF and IFN-γ production long after viral challenge (Mouat et al., 2022c). It is important to note that upon viral infections, ABCs have been reported to downregulate inhibitory receptors and checkpoints, such as Fas, CTLA4 or PD1, as well as regulatory cytokines (Mouat et al., 2022a), such as IL-10. Taken together, these lines of evidence seem to indicate that ABCs may shape the immune milieu towards a more ‘autoimmune’ prone state upon viral infections. In line with this, hepatitis C virus infection has been correlated with the emergence of an ABC-like population producing RF antibodies and inducing a Th1 shift (Comarmond et al., 2019). Type I IFN activation may bridge viral exposure and in ABCs expansion, also in connection with autoimmune phenomena. In fact, dysregulated activity of IFN regulatory factors in response to stimulation with IL-21 may account for enhanced ABCs formation in mouse models of lupus (Manni et al., 2018), thereby reinforcing the functional link between viral exposure and autoimmunity occurrence.

Recent evidence using the CIA model has demonstrated that ABCs were also expanded shortly after arthritis onset in the absence of viral infections. Interestingly, ABCs were expanded in spleen and joint tissues, as well as in blood (Qin et al., 2022). *In vitro* studies demonstrated the central role of IL-21 and TLR7 signalling in driving ABCs differentiation in CIA immunized mice (Qin et al., 2022). Furthermore, cholesterol accumulation in CD11c^+^ B-cell has been reported to trigger autoimmune diseases in mouse models (Ito et al., 2016). Interestingly, altered cholesterol metabolism, lipoprotein dysfunction and accelerated atherogenesis are common hallmarks of autoimmune diseases (McMahon et al., 2006; Charles-Schoeman et al., 2012; Ronda et al., 2014; England et al., 2018), including RA, and shared mechanisms have been postulated (Weber et al., 2023). Taken together, mice studies indicate that ABCs expansion may contribute to the onset of autoimmunity in arthritis, either by autoantibody production via TLR stimulation, and/or skewing Th fate. Characterizing chemokine receptor profiles may help in understanding ABCs involvement at this stage, as ABCs usually display lower CXCR5 expression. Altogether, these lines of evidence reinforce previous studies from other autoimmune disease models (reviewed in (Ma et al., 2019)).

Despite the reassuring findings from animal models, data from human studies on the role of ABCs in the early stages of RA remain rather scarce (Figure 2), in part due to technical and analytical limitations. Growing evidence from the last decade has shaped our understanding on these stages, prompting a paradigm shift from a joint-centred to a secondary lymphoid organ-centred approach, as changes in the synovial are known to be subtle during this phase (Jiménez-Martínez et al., 2024; O’Byrne and van Baarsen, 2024). A number of alterations have been described in lymph nodes in this setting. Interestingly, a premature senescence profile has been described (De Jong et al., 2025), especially for T-cells, including a Th1 predominance and reduced regulatory T-cell counts (Ramwadhdoebe et al., 2016). Although less evidence is available from B-cell subsets, disturbed subsets have been demonstrated (Van Baarsen et al., 2013), and increased IL-21 production, elevated Tph frequencies at the lymph node level have been reported (Ramwadhdoebe et al., 2019; Anang et al., 2022). All these findings suggest that the earliest phases of RA are characterized by an immune microenvironment permissive for ABCs generation. In particular, the combination of Th1 bias, increased IL-21 production, and expanded Tph cells may provide the appropriate signals required for ABCs differentiation and activation, even before overt synovial inflammation becomes dominant. Unfortunately, direct analyses of ABCs in the lymph node microenvironment at this disease stage are still lacking. Recent evidence has demonstrated higher levels of circulating ABCs in clinically-suspect arthralgia individuals (Miranda-Prieto et al., 2025), thereby mirroring findings from animal studies. ABCs numbers were unrelated to clinical manifestations or autoantibody levels in CSA, likely suggesting that ABCs expansion is a common trait in this ‘at risk’ phase, rather than being associated with specific clinical features. Of note, reduced sample size prevented further analyses. Larger and prospective long-term studies are needed to gain knowledge about ABCs expansion in human individuals at this stage. Furthermore, paired (synovial + blood) analyses are warranted to better understand early disease mechanisms.

Studies in RA patient populations mostly came from established disease subsets (Table 1) and have helped to shed some light into the involvement of ABCs. However, different analytical platforms and terminology have challenged this field. Certain studies demonstrated a positive association with disease activity (Bao et al., 2022; Qin et al., 2022; Kim et al., 2025), hence pointing to a potential role in disease exacerbation. From a clinical perspective, a sub-analysis of a real-world cohort has revealed that ABCs expansion at onset was associated with poorer clinical responses (at 6 and 12 months) upon csDMARD initiation in recent-onset RA patients (Miranda-Prieto et al., 2025). These data pioneered the association between ABCs and treatment outcomes in RA, and highlights their potential role as predictive biomarkers. However, further evidence on their role as predictive biomarkers is still lacking.

Compelling evidence has demonstrated a positive association with proinflammatory cytokine levels and/or production, especially IFN-γ, IL-21 or TNF (Bao et al., 2022; Qin et al., 2022; Vidal-Pedrola et al., 2023; Miranda-Prieto et al., 2025). Furthermore, results from a recent study also demonstrated that ABCs paralleled Tph counts, similar to animal studies. Tph, and Tfh, may rhus represent potential sources of CD40L, IL-21 and IFN-γ, which may driver ABCs generation (Keller et al., 2021). Altogether, this reinforces a potential Tph/ABCs/IL-21 axis in this condition, which is also consistent with equivalent findings reported in juvenile idiopathic arthritis (JIA) (Fischer et al., 2022)). This observation also argues for a broader T-cell/B-cell pathogenic axis rather than a simple association between soluble cytokines and B-cell abundance. In RA, ABC expansion may reflect the integrated effect of T-cell polarization, including Tph/Tfh-derived IL-21 and IFN-γ, together with sustained antigen presentation and costimulation. The potential contribution of senescent T-cell subsets should also be considered, as chronic inflammatory activation may enhance helper functions while reducing immune regulation, thereby favouring persistence of ABCs and the inflammatory feedback loops they sustain, including B-cell/T-cell crosstalk. The exploitation of this axis as a therapeutic target may be conceivable (discussed later).

ABCs accumulation has been also registered at the synovial level in a number of studies in RA (Table 1) as well as in JIA (Dirks et al., 2021). In fact, ABCs have been found to be at 10 times higher frequency in synovial fluid compared to blood in RA patients (Qin et al., 2022). This observation aligns with gene expression profiles related to chemotaxis and migration-related pathways. Mounting evidence from mice studies have proposed a role for CXCR3 in this setting (reviewed in (Mouat et al., 2022b)). Interestingly, CXCR3 has been reported to be upregulated in ABCs in mice and humans, despite being absent in other B-cell subsets. An elegant series of experiments have recently unveiled a connection between ABCs and stromal pathogenesis at the synovial level. By combining mass cytometry and single-cell RNA sequencing, ABCs expansion in RA synovium was confirmed, also demonstrated certain subset heterogeneity which may implicate tissue-specialized pathogenic mechanisms (Zhang et al., 2019). More recently, by using ABCs-conditioned media, Qin and coworkers demonstrated that ABCs were able to induce fibroblast-like synoviocytes (FLS) activation, via upregulation of adhesion molecules, proinflammatory cytokines and matrix-metalloproteinases *in vitro* (Qin et al., 2022). Furthermore, ABCs promoted the upregulation of IFN-stimulated genes in FLS, hence leading to an aggressive phenotype and synovitis perpetuation. This effect was attributed to the activation of TNF-mediated ERK and JAK-STAT1 pathways, and specific signalling blockade led to the abrogation of FLS activation, thus opening new avenues for treatment. Altogether these findings are relevant in a two-fold manner. First, as a direct crosstalk between ABCs and FLS activation is established, despite this phenomenon has been classically attributed to T-cells, whereas the role of B-cell subsets have been neglected. Second, as it demonstrates an active participation of ABCs in driving RA progression *in situ* at synovial level, and not just in the orchestration of RA immunopathogenesis at distant lymphoid compartments.

### ABCs as drivers of disease comorbidity

**FIGURE 3:**
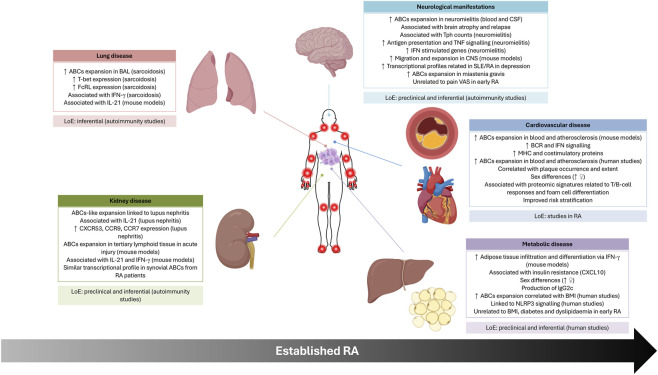
Putative roles of ABCs in driving comorbidity in RA. This figure summarizes current evidence supporting tissue-spanning effects of ABCs expansion and activation across tissues and organs on RA-associated frequent comorbidities. ABCs (center) may traffic to or interact with multiple organs—including vasculature, lung, central nervous system, adipose tissue, and other anatomical sites, where they exert context-dependent pathogenic or immunomodulatory functions. Mechanistic pathways (indicated in the boxes) include pro-inflammatory cytokine production, antigen presentation and T-cell polarization, autoantibody and immune-complex formation, promotion of ectopic lymphoid structures, altered signalling and phenotypes, and metabolic/inflammaging circuits. Evidence tags indicate whether support derives from RA studies or from related autoimmune or cardiometabolic conditions. Collectively, these data support ABCs as systemic effectors linking joint autoimmunity with multi-organ comorbidity. Level of evidence is indicated at the bottom of each box, and further explained in Table 2.

Inflammation and immune dysregulation are central to the development of RA comorbidities, which are also strongly associated with aging (Santos-Moreno et al., 2021). This overlap suggests that immunosenescence and inflammaging may act as shared upstream mechanisms in comorbidities, promoting endothelial dysfunction, impaired tissue repair, and a persistent pro-inflammatory milieu that accelerates organ damage beyond the joints (Bauer, 2020; Santoro et al., 2021; Weyand and Goronzy, 2025). In this context, age-related immune remodelling may favour the impairment of immune resolution and tissue homeostasis, which may help explain why comorbidities in RA often mirror features of accelerated biological aging (Bauer, 2020; Santoro et al., 2021). Although the actual immune circuits remain unclear, ABCs emerge as plausible contributors to RA comorbidity burden (Figure 3). Moreover, data supports the role of tissue-resident or tissue-homing Tph-like cells in sustaining ABCs recruitment and local differentiation in distant organs. Importantly, direct evidence for certain outcomes is still sparse in this setting (Figure 3) (Table 2). Evidence may be inferred from other, related contexts, although the translation of findings requires great caution, and avenues for further research on the role of ABCs involvement in RA comorbidities are identified and discussed (Table 2).

**TABLE 2:** Summary of the evidence on ABCs and comorbidities in RA.

Comorbidities	Preclinical evidence	Evidence from human studies	Evidence in RA	Main gaps
Cardiovascular disease	☑ (non-RA models)	☑☑☑ (non-RA)	☑ (early RA)	• Prospective studies • Clinical validation • Calibration and role as a biomarker
Lung disease	☒	☑ (autoimmune diseases)	☒	• Mechanistic evidence in mouse models, including autoimmune-prone • Systemic and tissue level analysis in RA with ILD • Association with immunosenescence features in RA
Neurological manifestations	☑ (non-RA models)	☑☑ (autoimmune and non-autoimmune diseases)	☒	• Mechanistic evidence in autoimmune mouse models, especially regarding pain • Systemic and tissue (?) level analysis in RA with neurological/CNS manifestations, including fatigue • Association with Tfh and type I IFN circuits in RA at tissue level
Kidney disease	☑ (non-RA models)	☑ (autoimmune diseases)	☒	• Mechanistic evidence in RA mouse models • Systemic and tissue level analysis in RA with kidney disease • Association with immunosenescence features in RA • Role of IL-21 and IFN-γ in driving ABCs expansion at local level
Metabolic disease	☑ (non-RA models)	☑ (non-autoimmune diseases)	☑/☒ (early RA)	• Mechanistic evidence in RA mouse models • Tissue level analysis in RA with metabolic disease • Role of metabolic features in driving ABCs expansion in RA

The analysis of studies evaluating the associations between ABCs, and comorbidities are summarized by organ system. The level of evidence is represented as ☑ or ☒, depending on the presence or absence of evidence existing on preclinical approaches (mouse models or *in vitro* platforms), human studies (with or without autoimmune backgrounds), or RA, populations. The number of symbols represents the amount of existing evidence, under a non-systematic review approach. The main research gaps that inform the research agenda for each organ system are summarized in the last column.

### Cardiovascular disease

Atherosclerotic cardiovascular disease (CVD) represents the first cause of mortality and morbidity in RA patients (England et al., 2018). CV risk excess in RA results from a combination of traditional risk facts and immune dysregulation, although mechanisms underlying the latter are far from being clear. B-cells are known to have key roles in atherosclerosis by producing antibodies against self and modified antigens, cytokine release and T-cell interactions. Moreover, immune aging is related to atherosclerosis, thus suggesting the involvement of ‘aged’ immune cells, such as ABCs, in this comorbidity. Importantly, atherosclerosis plaques possess all elements required for ABC generation, including modified self-antigens, necrotic cell–derived chromatin and nucleic acids, and local production of Th1 cytokines (Knox et al., 2025b).

Mouse models of atherosclerosis (Ldlr^−/−^ and Apoe^−/−^) demonstrated increased ABCs frequencies in mice with atherosclerosis compared to wild-type counterparts (Smit et al., 2023). ABCs accumulation was also observed at spleen and bone marrow, and correlated atherosclerosis burden (Pattarabanjird et al., 2023). Moreover, these ABCs showed higher expression of genes encoding MHC, costimulatory and inhibitory proteins, also confirmed by pathway analysis, hence suggesting a functional role (Smit et al., 2023). Equivalent results were observed in a different mouse model (PCSK9-encoding adenovirus inoculation), revealing ABCs accumulation in secondary lymphoid organs (spleen), along with a proinflammatory and specific gene expression profiles observed in ABCs (de Mol et al., 2025). Additionally, ABCs were expanded in the spleen in Apoe^−/−^ mice even without being exposed to a high-fat diet. T-bet deletion in Apoe^−/−^ reduced the ABCs number without any significant impact on other B-cell populations, diminished aortic lesion area in comparison with wild-type littermates, and caused a shift in antibody production (Knox et al., 2025a). Altogether, these findings support the crucial role of ABCs in atherosclerosis in mice (non-RA models), and shed new light into potential functional mechanisms. Interestingly, ABCs may also contribute to explain the differences between sexes in atherosclerosis, as females not only exhibited higher ABCs frequencies, but also an enhanced activation status based on specific gene expression signatures (Smit et al., 2023). Studies on mice models of stroke supported these results (Korf et al., 2022), thus supporting the involvement of ABCs across atherosclerosis-affected tissues. However, no evidence in RA or autoimmune mouse models is available (Table 2).

Increased ABCs have been also documented in non-RA studies in humans linked to coronary artery disease severity (Pattarabanjird et al., 2023), also exhibiting a higher activation status. Interestingly, divergent pathways were found to hallmark ABCs compared to DN2, hence highlighting the differences between these two subsets. Interestingly, BCR, HMGB, and type I IFN signaling predominated in the latter. A recent study demonstrated that ABCs were also present in non-RA human atheroma plaques, in higher frequencies than in PBMCs, and showing an enhanced T-bet expression. These results may be explained by the proinflammatory plaque milieu, which contains cellular debris and can cause constant B-cell activation and consequently, promoting ABC fate (Smit et al., 2023).

ABCs expansion in atherosclerosis has been also reported in the context of early RA. ABCs were found to correlate atherosclerosis occurrence and extent, and they were unrelated to classical risk factors, and other subclinical endpoints such as cIMT or vascular stiffness (Miranda-Prieto et al., 2025). Besides, no associations with lipoprotein levels were found. Furthermore, ABCs numbers paralleled proteomic signatures related to B-cell activation, T-B cell interaction, T-cell dependent B-cell activation, T-cell polarization and macrophage differentiation into foam cell (Miranda-Prieto et al., 2025), thus aligning with single-cell analysis and evidence from animal models. Interestingly, ABCs levels demonstrated significant clinical added value, as ABC strata improved risk stratification over conventional algorithms (SCORE), thus supporting their role as clinical biomarkers, either independently or by the combination of existing instruments. Experimental and clinical validation studies are needed to better understand relevance of these findings (Table 2).

### Lung disease

Around 30%–40% of RA patients suffer from pulmonary outcomes. Lung involvement is an early manifestation of the disease, and a major determinant of quality of life and a leading cause of death in RA (Figus et al., 2021), also linked to increased CV risk (Clarson et al., 2020). Underlying mechanisms of respiratory comorbidities in RA are not fully understood, but chronic inflammation and immunoaging are thought to play a role (Figus et al., 2021).

ABCs levels have been studied in sarcoidosis patients, an autoimmune condition also linked to interstitial lung disease (ILD). ABCs-like (CD19^+^CD11c^+^CD21^low^) were expanded both in blood and in bronchoalveolar lavage (BAL) samples (Figure 3). ABCs in BAL outnumbered those of blood paired samples and exhibited a higher T-bet expression, hence mirroring findings from synovial-blood studies in RA (Phalke et al., 2020) as well as those from atherosclerosis in non-RA populations. Interestingly, ABCs also exhibited a specific expression profile of FcRL, as observed in RA synovia (Vidal-Pedrola et al., 2023), thus reinforcing this point. Enhanced IFN-γ production in sarcoidosis is thought to promote ABCs in this condition (Phalke et al., 2020). Furthermore, animal models have demonstrated a promising role for IL-21 in this scenario (Um et al., 2024).

However, neither animal models nor studies in human RA patients are available (Table 2). Lung involvement in RA has been associated with T-cell senescence (Michel et al., 2007) and B-cell responses. ILD in RA has been associated with higher prevalence of disease-related (reviewed in (Guo et al., 2024)) and emerging autoantibodies (Harlow et al., 2013; Chen et al., 2015). Moreover, ectopic lymphoid structures have been documented at local tissue (Akiyama and Kaneko, 2022), thus suggesting *in situ* germinal center reactions contributing to fibrosis in lung parenchyma and resembling that of synovial processes. Of note, a range of pro-inflammatory cytokines have been reported, which may drive or interact with B-cell activation or migration events (Van Kalsbeek et al., 2023). However, the role of IFN-γ in this setting seems to be more complex, with a bi-phasic pattern being suggested (Gochuico et al., 2008; Solomon and Brown, 2012), although evidence is rather scarce. Although it may be tempting to speculate about the local ABCs expansion at the lung microenvironment, it must be noted that these lines of evidence are extrapolated from other disease contexts, and no studies have evaluated ABCs frequencies at systemic or BAL compartments in RA patients with ILD.

### Neurological manifestations

ABCs have been also linked to neurological manifestations in autoimmunity. Increased circulating frequency of ABCs has been reported in Neuromyelitis Optica Spectrum Disorder (NMOSD) patients (Figure 3), also correlating with clinical features including brain atrophy and relapse (Amano et al., 2024; 2025). ABCs levels paralleled those of Tph (Amano et al., 2024), as previously reported in other conditions. These ABCs displayed high expression of genes associated with enhanced antigen-presenting activity (FCGR2A, FCER1G and FCER1A) and TNF signaling pathway (TNFA*,* TRAF3 and TRAF6). Moreover, pathway enrichment showed enriched MHC II complex, chemotaxis and migration, and type I IFN pathways in ABCs from cerebrospinal fluid, suggesting an enhanced proinflammatory activity of ABCs at target tissue level (Zhang et al., 2021). Furthermore, ABCs have been also reported to be able to migrate to and expand within central nervous system (CNS) in mice models (Mouat et al., 2022b), although evidence in autoimmune models is lacking (Table 2). Collectively, these lines of evidence point to a role of ABCs in neurological manifestations likely via T-cell activation and differentiation (Amano et al., 2024), and potentially via crosstalk with stromal cells, as reported in mouse model of neuroinflammation (Korf et al., 2022). Evidence from human studies in miastenia gravis (MG) support this notion. ABCs were associated with disease activity, despite not showing correlation with autoantibody titres (Lu et al., 2025), likely suggesting mechanisms other than antibody production in MG, such as cytokine production or antigen presentation.

Furthermore, dysregulated B-cell profiles have been reported in major depression, linked to altered immunological synapse formation, antigen processing and presentation, and BCR signalling (Yin et al., 2025). Mice models demonstrated increased expression of genes linked to ABCs biology (TBX21, NLRP3) and enrichment in transcriptional profiles shared with SLE and RA (Yin et al., 2025) (Figure 3). Higher T-bet expression in ABCs expanded in spleen, and clinical and immunological findings strengthened these findings (Yin et al., 2025).

The potential role of ABCs in neurological/CNS manifestations of RA remains largely unexplored, even in animal models. From a mechanistic point of view, ABCs may be hypothesized to contribute to neurological manifestations or fatigue via systemic inflammation, as well as to pain via antibody production and inflammatory mechanisms. Interestingly, autoantibodies may contribute to pain sensitization independent of overt inflammation, especially in early or preclinical RA stages (Wigerblad et al., 2016). A recent study did not find associations with pain or other related manifestations (Miranda-Prieto et al., 2025), although more studies are needed, also including other outcomes such as depression spectrum disorders. Moreover, whether they play a role in fatigue remains known, despite representing a major and predominant symptom in RA.

### Kidney disease

Kidney disease is a common comorbidity of RA, and also linked to premature aging and immunosenescence. Solid evidence has demonstrated an association between higher disease activity and accelerated kidney function decline (Fukui et al., 2025; Ausserwinkler et al., 2026), thus pointing to potential shared pathogenic mechanisms.

ABCs have been involved in kidney damage and disease in autoimmunity, namely, in SLE. DN B-cells, including the ABCs subset (Figure 1), have been associated with lupus nephritis (LN) severity, (Figure 3), and reduced numbers have been proposed as a biomarker of LN remission (You et al., 2020). DN displayed a differential chemokine profile (CCR9, CCR7, and CXCR5), which may be associated to tissue homing to inflamed tissues. Of note, DN numbers correlated IL-21 levels in this study (You et al., 2020), likely pointing to an ABCs bias. Data from lupus mouse models supported these findings (Nickerson et al., 2023). ABCs expansion in human patients with LN has been associated with lower levels of P2RY8 downregulation (He et al., 2021), which in turn is related to TLR7 activation. P2RY8 dysregulation has been also related to enhanced migration properties of B-cells and altered chemokine expression and impaired checkpoint regulation, especially in the context of extrafollicular responses (He et al., 2021). More recent evidence has revealed the involvement of an ABC-like population in SLE patients, in relation to LN and disease activity, and linked to metabolic and functional dysregulations (Wu et al., 2019). Interestingly, *in vitro* evidence demonstrated a potential role as therapeutic targets.

ABCs have been also linked to acute kidney injury, via tertiary lymphoid tissue (TLT). ABCs accumulate in TLT after kidney injury and have a key role in TLT expansion (Sato et al., 2022). Local senescent T-cells provide IL-21 and IFN-γ to ABCs, favoring their activation and thus TLT expansion (Sato et al., 2022). TNF superfamily members, mainly TNFSF8 and TNFRSF8, orchestrate the crosstalk between ABCs and T-cells (Sato et al., 2022). Interestingly, scRNA-Seq analyses have demonstrated increased expression of these mediators in immune cells present in synovial tissues from RA patients, and ABCs exhibited the largest TNFRSF8 expression among B-cell subsets (Sato et al., 2022), thus pointing to a shared mechanisms between kidney and joint damage.

Whether humoral responses underlie kidney disease in RA remains largely unexplored, also in mouse models of the disease. Although a certain role may be expected in immune complex–mediated glomerulonephritis or rheumatoid vasculitis with renal involvement via antibody production, evidence is scarce for other forms of kidney disease. By virtue of their connection with disease activity exacerbation in RA, ABCs may potentially contribute by amplifying systemic inflammation and propagating immune responses upon T-cell activation. However, human studies focused on ABCs expansion in kidney disease in RA are not available, either at systemic or local tissue levels (Table 2).

### Metabolic disease

Senescence and inflammaging and autoimmunity are known to promote metabolic disorders, such as obesity and endocrine diseases tissues (Hägglöf et al., 2022; Liu et al., 2023).

Enhanced immune cell infiltration and autoantibody production has been reported in adipose tissue in obese individuals (Frasca et al., 2021b). Animal studies have demonstrated that ABCs can be also expanded in visceral adipose tissue in mice (Frasca et al., 2020). *In vitro* approaches revealed that adipocytes may promote enhanced differentiation from follicular B-cells into ABCs, probably via release of proinflammatory cytokines (IFN-γ, TNF and IL-6) or chemokines (CCL10, CCL2 and CCL5) (Figure 3) (Frasca et al., 2020). Moreover, fatty acids secreted in the adipose tissue have shown to stimulate ABCs differentiation by T-bet activation, recognition of adipocyte derived-specific antigens by BCR, recognition of fatty acids by TLRs and an increase in cytokine receptors like IFN-γ receptor (Frasca et al., 2021b; 2023). ABCs were not only expanded in adipose tissue but also established functional crosstalk with other infiltrating immune cells. Further mechanistic evidence identified iNKT as a central player guiding ABCs differentiation and antibody production, via IFN-γ production.

Animal models have also demonstrated that ABCs (CD11c^+^T-bet^+^ B-cells) can cause insulin resistance, by producing CXCL10 upon TLR stimulation (Hägglöf et al., 2022). CXCL10 and other B-cell factors have been previously described to worsen T2D and metabolic disease (Schulthess et al., 2009). Moreover, CXCL10 secretion by ABCs was also found to stimulate weight gain during obesity in mice (Hägglöf et al., 2022). Interestingly, these effects were restricted to females, whereas male individuals exhibited a modest ABCs accumulation in the spleen and reduced effect on glucose tolerance, thus suggesting the interplay with sex factors during obesity. Moreover, ABCs ablation by genetic deletion of TBX21 also suggested a causal role for IgG2c antibody production, which was connected with a divergent composition of macrophage pools in adipose tissue in mice lacking ABCs (Hägglöf et al., 2022). Consequently, mice lacking T-bet in B-cells were protected from metabolic symptoms of obesity, although diseased phenotype could be induced by serum transfer experiments (Hägglöf et al., 2022). Altogether, these findings demonstrate that the role of ABCs in obesity is more complex than initially conceived, with multiple, complementary mechanisms being involved.

ABCs-like cells were also reported to be expanded in adipose tissue samples from overweight and obese individuals, but not in their lean counterparts (Hägglöf et al., 2022). Interestingly, ABCs did not correlate age but BMI in these individuals, hence suggesting that BMI was the key factor in driving ABCs-like accumulation during obesity. Moreover, ABCs were shown to be expanded in fat-associated lymphoid clusters during aging as a consequence of IL-1 signaling through the NLRP3 inflammasome (Camell et al., 2019), suggesting another possible mechanism for ABCs expansion during obesity and, more importantly, reinforcing the relevance of systemic, other than local tissue, depots in obesity.

Recent evidence in RA patients (Miranda-Prieto et al., 2025) failed to demonstrate an association between circulating ABCs counts and obesity or waist circumference, and the same could be applied by other metabolic features such as diabetes or dyslipidaemia. Although obesity and adipose tissue inflammation are key hallmarks of RA, it should be noted that immune-inflammatory mechanisms outcompete classical metabolic features in this condition, thus probably reducing their relative weight in RA as pathogenic mechanisms (Gonzalez et al., 2008; Symmons and Gabriel, 2011). Similarly, whereas studies in RA have focused on blood and joint compartments, other tissues (such as adipose depots) have been neglected (Table 2), despite of the growing interest on their immunological relevance.

## ABCs in RA: A potential tool for personalized medicine?

From a translational perspective, ABCs may represent a promising cellular hub linking autoimmunity, chronic inflammation and immunosenesce to RA progression and multimorbidity. This paves the ground for translational and therapeutic targeting of ABCs in RA.

Recent studies in RA suggesting an early ABCs expansion open the question whether they may hold value as disease biomarkers. As they may reflect underlying immune remodelling before overt clinical manifestations, whether ABCs could serve as diagnostic biomarkers require dedicated studies, although single biomarkers are unlikely to contribute to diagnosis-decision in complex disease settings. Current evidence may point to a role as predictive biomarkers to csDMARD (Miranda-Prieto et al., 2025), probably linked to aggressive disease trajectories or B-cell-driven phenotypes. Moreover, evidence has proved a role for ABCs as biomarkers of atherosclerosis in RA, also demonstrating incremental clinical value over existing instruments. Although these results require clinical validation and calibration in long-term (Table 2), prospective studies, it encourages the assessment of ABCs as stratification biomarkers, especially in preclinical RA or early disease stages where biological heterogeneity is substantial and clinical endotypes are still evolving. However, clinical translation will require standardized phenotypic definitions, harmonized platform strategies, and prospective validation across independent cohorts. In parallel, ABCs may be useful as biomarkers of immune aging, which may help to escape from the T-cell-centered plateau, especially in research applications.

On the other hand, ABCs may also emerge as potential therapeutic target in this setting. Their reliance on TLR7/TLR9 signaling, type I IFN, and IL-21 suggests that interventions targeting upstream inflammatory circuits may indirectly limit ABCs expansion or pathogenic activation. Evidence on B-cell depletion therapies in RA has demonstrated that clinical benefit may not parallel changes in antibody responses, hence pointing to the role of other mechanisms such as antigen presentation or cytokine production. At the functional level, ABCs are well equipped with cellular machinery to promote these processes, compared with other B-cell subsets. Interestingly, B-cell depletion therapy has led to a reduction in ABCs in SLE patients, but not on Tph/Tfh subsets (Wortel et al., 2021). Contemporary evidence on other markers, such as adenosine receptor 2a, or nanomedicine approaches, such as polymer-coated gold nanoparticles, may built the therapeutic prospects of ABCs in coming years (Hočevar et al., 2022; Levack et al., 2022). Of note, preclinical models and human studies have demonstrated an increased activation of type I IFN pathway in ABCs. Elucidating the molecular drivers of type I IFN upregulation on ABCs expansion and their functional consequences may provide new clues on the pathogenesis and clinical value of IFN assays (Rodríguez-Carrio et al., 2023b; 2023a), and it may open a new therapeutic rationale through the use of type I IFN targeted strategies.

Finally, the role of T-cell/B-cell crosstalk in establishing ABCs pathogenicity may also open new therapeutic avenues in RA. In this regard, it is tempting to speculate that T-cell co-stimulation blockade may also affect ABCs differentiation and activation. Thus, abatacept, by blocking CD80/CD86-mediated T-cell activation, may indirectly attenuate the helper signals that sustain pathogenic ABCs responses. Their beneficial effect on regulatory B-cell subsets (Carvajal Alegria et al., 2021) may also contribute to its therapeutic outcomes. Moreover, data is suggestive of a potential effect in memory B-cells (Gazeau et al., 2017). Evidence in established RA patients has demonstrated a sustained (14 and 24 weeks) decline in ABCs-like populations (CD19^+^CD11c^+^IgD^−^CD27^−^) upon abatacept initiation (Wang et al., 2025). Interestingly, ABCs-like frequencies emerged as a marker of clinical response. Furthermore, studies in JIA have also demonstrated that abatacept led to a modulation of CD21low B-cells (AitDowd et al., 2025), also in association with Tfh and related T-cell subsets, hence strengthening the relevance of the T-cell/B-cell crosstalk from a therapeutic perspective across inflammatory arthritidies. Such studies could be particularly informative in early RA, where T-cell/B-cell crosstalk may be more central to disease initiation than in established disease. Early ABCs expansion in RA may represent a strong therapeutic rationale in this scenario. Although direct evidence on the impact of abatacept on ABCs frequencies, phenotype, or function in early RA is still lacking. Clinical trials and mechanistic studies are warranted to test this hypothesis.

### Future perspectives and research agenda

Despite recent breakthroughs, important knowledge gaps remain (Table 2), which limit the translation of these advances into the clinical setting. The overarching research questions on ABCs relate to methodological and analytical domains. Overall, the precise ontogeny and functional heterogeneity of ABCs subsets in inflammatory arthritis are not fully resolved. Moreover, consensus markers for their identification across tissues and disease stages are lacking, and the identification of RA-specific phenotypical traits are awaited. Additionally, a better understanding of cellular phenotypes and origin will allow to gain understanding towards the complex relationship between cellular phenotypes and disease context, which represent a major unmet research need. Moreover, integrating aging biology frameworks with immunopathology circuits in preclinical models of RA may not only help to dissect their role in immunosenescence, as well as the role of premature aging-related immune remodelling in disease progression, but also to clarify whether ABCs represent a modifiable target to prevent or ameliorate disease burden. Future research should prioritize harmonized phenotyping strategies, single-cell and multi-omic approaches to dissect ABCs functional programs.

From a clinical standpoint, longitudinal studies in preclinical and early arthritis are particularly scarce, thus limiting our understanding of temporal dynamics and causality, although current evidence hold promise on their role as biomarkers and, potentially, therapeutic targets. Prospective cohort studies spanning preclinical to established disease are required to shed new light into the actual roles of ABCs in disease natural history, including prognosis and prediction of response. Furthermore, the contribution of ABCs to extra-articular manifestations and comorbidity development remains largely inferential in most cases and requires mechanistic validation in the setting of RA. Exploration of inferential evidence from related disease contexts will help to identify shared mechanisms across diseases, which may in turn inform drug repurposing or treatment decisions.

Collectively, gaining understanding towards the role played by ABCs in RA may open new avenues for precision medicine into the triggering, progression and clinical manifestations of RA, especially in the earliest stages. A thorough assessment of their incremental value as biomarkers for disease stratification to identify high risk populations requiring a targeted approach may be proposed. However, the integration of ABCs data into existing clinical algorithms requires great caution in the absence of solid methodological frameworks to integrate translational biomarkers.

## Conclusion

ABCs have emerged as a compelling link between immunosenescence and autoimmunity, placing them as potential key players in RA pathogenesis, as they impact a wide spectrum of immunological phenomena such as inflammation, antigen presentation and antibody production. This cellular population emerges as a key player to understand how premature aging relates to disease mechanisms, with a focus on the B-cell compartment, which has been neglected compared to their T-counterparts. ABCs may thus be a cellular link between aging-related immune remodelling and the RA continuum, rather than a passive, innocent bystander of aging. Accumulating evidence has demonstrated a connection between ABCs expansion and RA, particularly in established disease. Scientific evidence in this field is fragmented, in part due to the lack of harmonization in experimental and methodological platforms and non-standardized nomenclature, thereby preventing a comprehensive understanding of the global picture.

The present review provides an integration of these findings into a relevant knowledge framework: the continuum of inflammatory arthritis. This longitudinal perspective may help to gain insight into the relevance of more recent evidence both from human studies and preclinical models has demonstrated that ABCs may prematurely accumulate in RA, and play a direct role in joint pathology. Furthermore, the pro-inflammatory and metabolically active phenotype of ABCs raises the possibility that they participate in systemic processes that underlie comorbidity occurrence, which has been largely neglected in ABCs studies. In this context, ABCs may represent a cellular substrate linking accelerated immune aging with clinical expression and multimorbidity in RA. Latest evidence from early RA studies strengthen the role of ABCs in disease processes, and points to a potential use as disease biomarkers. By integrating findings from RA and related inflammatory settings, we propose that ABCs may represent a shared immunopathogenic axis connecting chronic inflammation, immune ageing, and end-organ damage. Furthermore, ABCs may also provide mechanistic insight into sex differences in RA, including comorbidity development. However, a potential causal role in the latter should be considered with great caution, especially in the absence of studies generated in the RA setting. Based on the evidence from related conditions and pre-clinical evidence, ABCs hold promise to drive disease comorbidity in RA, and studies to decipher specific pathogenic circuits in RA are urged.
